# ARID1A Mutation in Metastatic Breast Cancer: A Potential Therapeutic Target

**DOI:** 10.3389/fonc.2021.759577

**Published:** 2021-11-04

**Authors:** Xuan Cheng, Jian-Xiong Zhao, Feng Dong, Xu-Chen Cao

**Affiliations:** ^1^ The First Department of Breast Cancer, Tianjin Medical University Cancer Institute and Hospital, National Clinical Research Center for Cancer, Tianjin, China; ^2^ Key Laboratory of Cancer Prevention and Therapy, Tianjin, China; ^3^ Tianjin’s Clinical Research Center for Cancer, Tianjin, China; ^4^ Key Laboratory of Breast Cancer Prevention and Therapy, Tianjin Medical University, Ministry of Education, Tianjin, China; ^5^ Department of Neurosurgery, Tianjin Medical University General Hospital and Laboratory of Neuro-Oncology, Tianjin Neurological Institute, Tianjin, China; ^6^ State Key Laboratory of Experimental Hematology, The Province and Ministry Co-sponsored Collaborative Innovation Center for Medical Epigenetics, Tianjin Key Laboratory of Cellular Homeostasis and Human Diseases, Department of Cell Biology, Tianjin Medical University, Tianjin, China

**Keywords:** ARID1A, metastatic breast cancer, SWI/SNF complex, endocrine resistance, synthetic lethality, therapeutic targets

## Abstract

Distant metastasis is the principal cause of mortality for breast cancer patients. Targeting specific mutations that have been acquired during the evolution process of advanced breast cancer is a potential means of enhancing the clinical efficacy of treatment strategies. In metastatic breast cancer, *ARID1A* is the most prevalent mutation of the SWI/SNF complex, which regulates DNA repair, recombination, and gene transcription. The low expression of *ARID1A* is associated with poor disease-free survival and overall survival of patients with luminal A or HER2-rich breast cancer. In addition, *ARID1A* plays a prominent role in maintaining luminal characteristics and has an advantage for identifying responses to treatment, including endocrine therapies, HDAC inhibitors and CDK4/6 inhibitors. The therapeutic vulnerabilities initiated by *ARID1A* alterations encourage us to explore new approaches to cope with *ARID1A* mutant-related drug resistance or metastasis. In this review, we describe the mutation profiles of ARID1A in metastatic breast cancer and the structure and function of ARID1A and the SWI/SNF complex as well as discuss the potential mechanisms of ARID1A-mediated endocrine resistance and therapeutic potential.

## Background

Breast cancer has become the most frequently occurring malignancy worldwide, with 2.3 million women diagnosed with breast cancer in 2020 (1). Despite advances in early diagnosis and comprehensive therapeutic regimens, 20–30% of breast cancer patients who are diagnosed with new or recurrent advanced-stage or metastatic breast cancers contribute to 90% of cancer-related deaths (2, 3). Because of their high heterogeneity, aggressive metastatic breast cancers have variable responses to treatment and different patient prognoses. The common distant metastatic organs are bone, lung, liver, and brain, with 5-year overall survival rates of 22.8, 16.8, and 8.5% and extremely short survival rates which are lower than 80%, the 5-year overall survival rate of breast cancer patients without metastasis (4).

Genetic alterations with clinical significance in advanced breast cancer are more abundant and more complicated than those in early-stage breast cancer (5). Massive parallel sequencing of 8,654 advanced breast cancers showed that 80.4% of the tumors harbored a genetic variation in at least one pathway with therapeutic implications (6). Although some driver genes, such as *PIK3CA, TP53, ERBB2, ESR1, AKT1*, and *BRCA1*, are detected in some early breast cancers, these genes are more frequently altered in patients with distant metastasis (7). Moreover, several studies have further demonstrated that the accumulation of genetic alterations during the process of cancer evolution between primary tumors and metastases and the exposure to systemic therapy itself lead to endocrine/chemotherapy resistance of tumor cells, resulting in treatment failure or disease progression (4, 8, 9). Thus, it is important and urgent to develop and research targeted agents aimed at newly discovered driver aberrations to guide personalized therapy against metastatic breast cancer.

The SWItch mating type/Sucrose Non-Fermenting (SWI/SNF) family, a member of chromatin remodeling complexes which include four distinct families in eukaryotes, was initially identified in *Saccharomyces cerevisiae* and is evolutionarily conserved, particularly among the ATPase subunits (10). SWI/SNF complexes utilize the energy of ATP hydrolysis to remodel chromatin accessibility by means of nucleosome sliding or nucleosome ejection and insertion (11). The bromodomain of ATPase subunits assists in targeting acetylated histones for SWI/SNF complexes (12). The AT-rich interactive domain (ARID) subunits are crucial for SWI/SNF-mediated chromatin remodeling and recruit SWI/SNF complexes to chromatin *via* nonselective DNA binding activity (13–15) or through interacting with other transcription factors. These complexes modulate multiple cellular processes and are associated with gene transcription and DNA repair. It is worth noting that mammalian SWI/SNF complexes contribute to not only activation but also repression of transcription. Brahma-related gene 1 (BRG1) and homologous Brahma participate in both transcriptional activation and repression of ER-driven genes in ligand- and context-dependent reprogramming (16–18).

A high prevalence of mutations in SWI/SNF chromatin remodeling complexes has been found in 20–25% of all human cancers (19, 20). Most of the mutations result in decreased protein expression of the SWI/SNF subunit, which influences subsequent chromatin changes and further transcription outcomes (10). The tumor suppressor AT-rich interactive domain protein 1A (*ARID1A*) is the most commonly mutated gene in SWI/SNF complexes. *ARID1A* is located on chromosome 1p35.11 and encodes a protein of approximately 250 kD. ARID1A is localized in the nucleus and has a broad tissue distribution. ARID1A is characterized by a 100-amino acid ARID domain conserved in eukaryotic organisms. The ARID domain is important for mediating ARID1A-associated SWI/SNF family DNA binding. It has been reported that the ARID domain does not preferentially interact with the AT-rich sequence, but it can recognize the pyrimidine-rich sequence of the β-globin locus (21). In addition, some LXXLL motifs in the C-terminal region of ARID1A are critical for ARID1A–glucocorticoid receptor (GR) interaction and subsequent GR-mediated transcription (21). Specific vulnerabilities attributed to the loss of tumor suppressors in tumor cells often create potential targets for therapy. *ARID1A* is one of the few genes that are mutated in pre-existing genomically unstable cancer cells, and the importance of *ARID1A* mutations is underestimated.

ARID1A is now known to have the highest mutation incidence, up to 46–57%, in ovarian clear cell carcinoma (22, 23). Several reviews have summarized the synthetic lethal strategies for ARID1A mutation (24, 25), but few review articles are available specifically in the context of metastatic breast cancer. Here we review the current understanding of ARID1A, including mutation profiles, prognostic values, and biological functions in metastatic breast cancer, discuss how ARID1A mutation mediates drug resistance, and summarize the potential therapeutic strategies for ARID1A-mutated metastatic breast cancer to curb this dreaded disease.

## *ARID1A* Mutation and Prognostic Values in Breast Cancer

**Figure 1:**
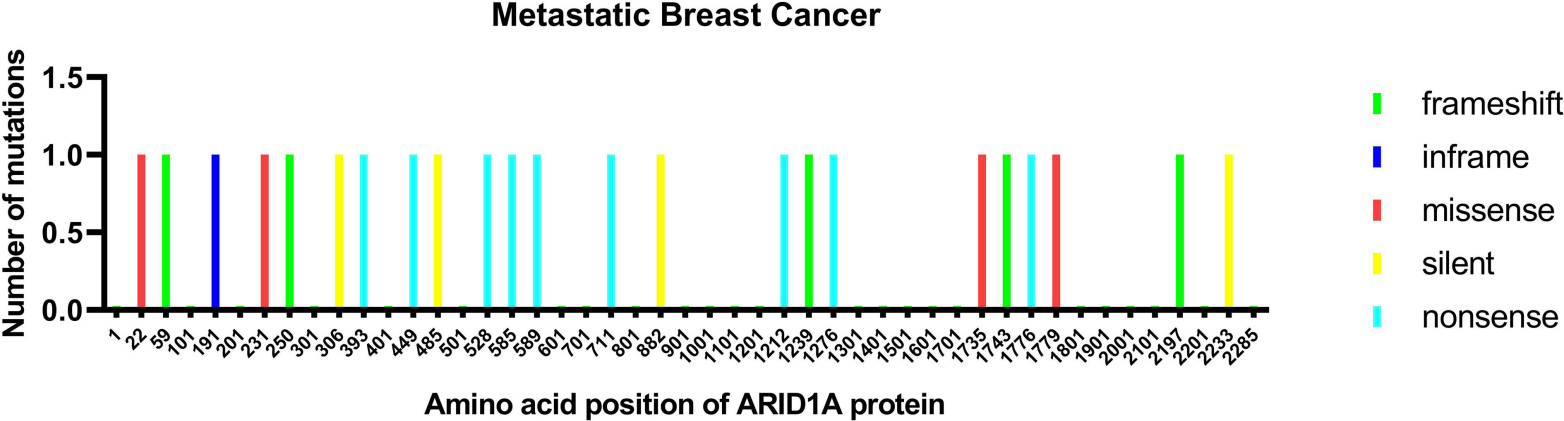
ARID1A protein mutation in metastatic breast cancer. The mutations are colored by the type of mutations: missense, nonsense, silent, and inframe.

In breast cancer, ARID1A is regarded as a tumor suppressor (26) and cooperates with CEBPα to suppress cell proliferation and migration (27). Inactivating *ARID1A* mutations were only present in 2.5% of all breast cancers. Nevertheless, the aberration frequency increased up to 12% in metastatic breast cancers with treatment resistance and 10% in inflammatory breast cancer (IBC), which has a higher tumor mutation burden than non-IBC (19, 28). Recently, in the search for newly emerging driver genes in metastatic breast cancer *via* whole-genome sequencing, Yates et al. found that genes related to the SWI/SNF family, especially *ARID1A*, were commonly wild type in primary breast cancer but inactive in recurrent breast cancer (29). Corresponding ARID1A alterations are shown in **Figure 1**.

Previous studies support *ARID1A* deletion as an independent prognostic factor for invasive breast cancer (30–32). The low mRNA expression of *ARID1A* is related to shorter overall survival in luminal A and human epidermal growth factor receptor 2 (HER2)-rich breast cancer (31). Takao et al. analyzed the immunohistochemical staining to evaluate the relationship between the downregulation of ARID1A and poor disease-free survival (33). Moreover, *ARID1A* may have a high prognostic value for drug sensitivity. Xu et al. verified a vital function of ARID1A in breast luminal lineage maintenance, which further revealed that the expression of ARID1A in the luminal type was higher than that in the nonluminal type (31, 32). The loss of *ARID1A* was more general in post-endocrine therapy metastatic cancer and regarded as the top candidate whose loss indicated fulvestrant resistance (32, 34). In addition, *ARID1A* loss or downregulation drives paclitaxel resistance and HER2/PI3K/mTOR-targeting drug resistance in breast cancer (35, 36). Therefore, ARIDIA is closely related to tumor metastasis or drug resistance-mediated tumor recurrence.

## Mechanisms Underlying the Therapeutic Responses of ER^+^ Breast Cancer

Estrogen receptor-positive (ER^+^) breast cancer is the most common breast cancer subtype. Endocrine therapy, as the mainstay of ER^+^ breast cancer treatment, can disrupt the interaction of estrogen with the ER ligand binding domain and inhibit the ER signaling pathway either by selective ER modulators/degraders (SERMs/SERDs) antagonizing ER or by aromatase inhibitors (AIs), which decreases the level of systemic estrogen by blocking the conversion of androgens to estrogens. While these treatments markedly reduce the risk of recurrence and death in ER^+^ breast cancer patients (37–39), resistance to endocrine therapy remains a major challenge (40).

Razavi et al. found that ARID1A loss-of-function mutations were acquired following treatment with endocrine therapy (41). A recent study has shown that ARID1A is indispensable for the activity of both tamoxifen (SERM) and fulvestrant (SERD), and the subsequent repression of ER target gene transcription is dependent on specific enhancers. Nagarajan et al. found that ARID1A relies on the FOXA1 protein to bind to the regulatory elements of chromatin (28). Tamoxifen-bound ER is enriched and binds to the FOXA1-dependent ARID1A binding site with an ARID1A-containing SWI/SNF complex to suppress gene expression under tamoxifen treatment. This is not associated with chromatin accessibility but with histone deacetylase 1 (HDAC1) recruitment. ARID1A loss promotes bromodomain-containing protein-4 (BRD4)-mediated transcription on account of reduced HDAC1 binding and increased histone H4 acetylation (H4ac) levels. Indeed ARID1A deletion makes breast cancer cells sensitive to bromodomain and extraterminal domain (BET) inhibitors and HDAC inhibitors. This provides rational treatment strategies for inactivated ARID1A-mediated endocrine resistance in breast cancer (28). Chidamide (an oral HDAC inhibitor with subtype specificity for the inhibition of HDAC1, HDAC2, HDAC3, and HDAC10) combined with exemestane (AI) improved the progression-free survival in advanced HR+ HER2- breast cancer patients who progressed after a previous endocrine therapy compared with placebo plus exemestane (42).

Furthermore, in ER^+^ breast cancer, ARID1A was found to participate in the maintenance of luminal identity by modulating cellular plasticity (32). ARID1A loss mainly results in the loss of chromatin accessibility, which tends to be in intergenic or intragenic regions. In addition, the recruitment of master luminal transcription factors such as ER, forkhead box protein A1 (FOXA1), and GATA-binding factor 3 (GATA3) and histone modification H3K27ac levels at correspondingly accessible sites is clearly decreased, impacting enhancer activity and ER-dependent transcription. Meanwhile, TEA domain transcription factor 4, which is enriched in basal-like cells, is recruited to accessible sites, indicating the switch of phenotypes from the luminal type to the basal-like type in ARID1A-loss breast cancer. The lack of ER expression in *ER*-positive breast cancer is related to endocrine-resistant therapy and *ER*-negative metastatic relapse (43). Hence, it is plausible that inactive ARID1A mutants are found in endocrine-resistant breast cancer.

Dysregulated cellular proliferation is a key hallmark of cancer, and targeting cell proliferative signaling is a widely used strategy for tumor treatment (44). In the cell division cycle, cyclin-dependent kinase-4 (CDK4)- and CDK6-mediated retinoblastoma phosphorylation is necessary to drive cells from G1 to S phase (45). Hence, drugs inhibiting enzymatic activities to induce cell cycle arrest have been used and proven to have significant efficacy in tumor therapy, including metastatic breast cancer (46, 47). As early as 2005, it was reported that ARID1A participates in cell cycle regulation and that its deletion induces impaired cell cycle arrest, indicating that ARID1A depletion may be associated with carcinogenesis (48). As mentioned above, inactivated ARID1A is related to endocrine resistance (28). An important mechanism of endocrine resistance is cell cycle dysregulation, which inhibits antiproliferative effects (49, 50). Based on the underlying mechanisms mentioned above and the remarkable outcomes of clinical trials, palbociclib or ribociclib (CDK4/6 inhibitor) combined with standard endocrine therapy is regarded as the first-line therapy for recurrent unresectable or metastatic ER^+^ breast cancer according to the NCCN guidelines (51–53). These findings indicate that CDK4/6 inhibitors are promising therapeutic strategies for ARID1A-mutated metastatic breast cancer.

## Promising Treatment Targets

**Figure 2:**
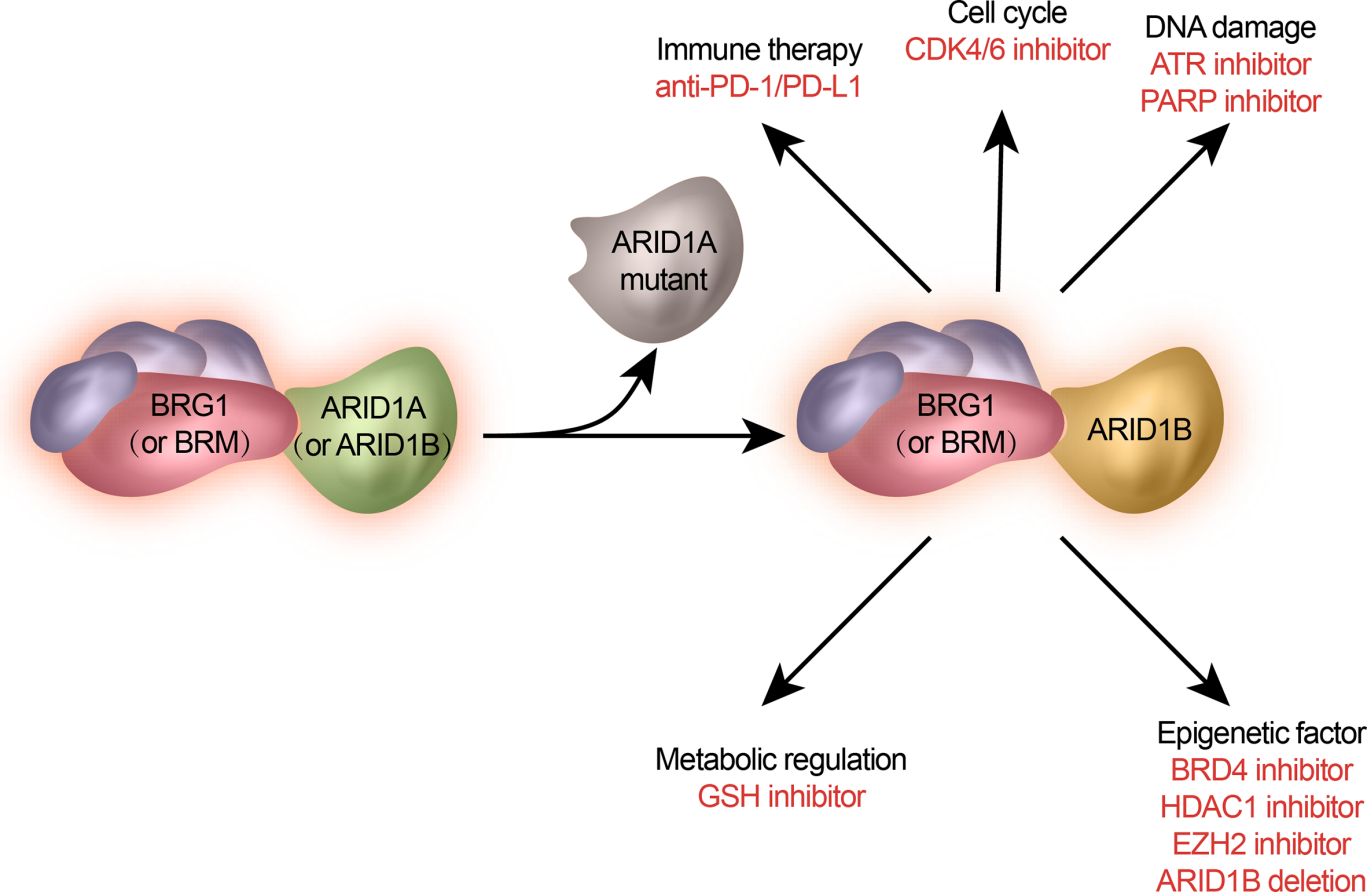
Potential treatments of ARID1A-mutated metastatic breast cancer. ARID1A mutation gives rise to specific signaling pathways and cellular functions. They are selected as therapeutic targets against ARID1A mutations.

Genetic mutations and altered molecular pathways serve as promising targets in personalized therapeutic approaches. Previous studies have revealed that *ARID1A* mutations can pre-exist in primary cancers and can be enriched during metastasis. Loss-of-function mutations in *ARID1A* often lead to the disruption of mammalian SWI/SNF complex integrity and subsequent transcriptional dysfunction. Therefore, *ARID1A* mutations simultaneously create an altered regulation of distinct target gene sets and generate a series of cellular dependencies or synthetic lethality. Here we summarize newly discovered potential therapeutic targets for metastasis- or endocrine-resistant breast cancer driven by mutated ARID1A (**Figure 2**) and the treatment approaches being tested in clinical trials involving patients with ARID1A-mutated cancer (**Table 1**).

**Table 1:** Clinical trials involving patients with ARID1A-mutated cancer.

Targets	Interventional agents	Clinicaltrial.gov ID	Disease setting	Study phrase	Estimated enrollment	Experimental setting	Study status
ATR	M4344	NCT02278250	Advanced solid tumors including an ARID1A-mutated cohort	I	98	M4344 single agent	Active, not recruiting
ATR	M6620	NCT03718091	Advanced solid tumors including an ARID1A-mutated cohort	II	30	M6620 single agent	Active, not recruiting
ATR	AZD6738	NCT03682289	Metastatic solid tumors (except clear cell ovarian cancer) including an ARID1A-mutated cohort (*n* = 39)	II	68	AZD6738 single agent	Recruiting
ATR	AZD6738	NCT04065269	Relapsed gynecological cancers with ARID1A loss or no loss	II	40	AZD6738 single agent; AZD6738 plus olaparib (PARPi)	Recruiting
PARP	Olaparib	NCT04042831	Metastatic biliary tract cancers including an ARID1A-mutated cohort	II	36	Olaparib single agent	Recruiting
PARP	Olaparib	NCT02576444	Metastatic solid tumors including an ARID1A-mutated cohort	II	64	Olaparib plus AZD5363 (Akti)	Active, not recruiting
PARP	Niraparib	NCT03207347	Any type of malignancy (except prostate) including an ARID1A-mutated cohort	II	57	Niraparib single agent	Suspended
BET	PLX2853	NCT03297424	Advanced malignancies including an ARID1A-mutated cohort	Ib/IIa	166	PLX2853 single agent	Recruiting
BET	PLX2853	NCT04493619	Advanced gynecological malignancies with ARID1A mutation	IIa	Less than 67	PLX2853 single agent	Recruiting
PD-1	Nivolumab	NCT04957615	Metastatic or surgically unresectable solid tumors with ARID1A mutation and CXCL13 expression	II	30	Nivolumab single agent	Not yet recruiting
PD-1	Nivolumab	NCT04953104	Metastatic or surgically unresectable urothelial cell carcinoma with ARID1A mutation	II	30	Nivolumab single agent	Not yet recruiting
PD-1	Toripalimub	NCT04284202	Metastatic NSCLC with ARID1A mutation	II	30	Toripalimub plus Dasatinib (multi-kinase inhibitor)	Not yet recruiting
PD-L1	pembrolizumab	NCT04633902	Unresectable or metastatic melanoma including an ARID1A-mutated cohort	II	41	Pembrolizumab plus olaparib (PARPi)	Recruiting
EZH2	Tazemetostat	NCT03348631	Recurrent ovarian or endometrial cancers including an ARID1A-mutated cohort	II	86	Tazemetostat single agent	Suspended
EZH2	CPI-0209	NCT04104776	Advanced urothelial carcinoma, ovarian clear cell carcinoma, endometrial carcinoma with ARID1A mutation	I/II	Less than 268	CPI-0209 single agent	Recruiting
EZH2	Tazemetostat	NCT05023655	Advanced or metastatic solid tumors with ARID1A mutation (except epithelioid sarcoma)	II	40	Tazemetostat single agent	Not yet recruiting
Others	Dasatinib	NCT02059265	Recurrent or persistent ovarian, fallopian tube, peritoneum, and endometrial clear cell carcinoma including a BAF250a expression loss cohort	II	35	Dasatinib (multi-kinase inhibitor) single agent	Active, not recruiting
Others	ENMD-2076	NCT01914510	Ovarian clear cell cancers including an ARID1A-mutated cohort	II	40	ENMD-2076 (multi-kinase inhibitor) single agent	Completed

Information on the clinical trials was obtained from https://clinicaltrials.gov on October 8, 2021.

ATR, ataxia-telangiectasia mutated and Rad3-related kinase; PARP, poly(ADP-ribose) polymerase; PARPi, PARP inhibitor; Akti, Akt inhibitor; BET, bromodomain and extraterminal domain; PD-1, programmed cell death protein 1; PD-L1, programmed cell death ligand 1; NSCLC, nonsmall cell lung cancer; EZH2, enhancer of zeste homolog 2.

### DNA Damage Repair—PARP and ATR

DNA damage response networks are important for the maintenance of genomic integrity especially in cancer cells. Cancer cells often undergo various exogenous and endogenous events, such as oxidative stress and replication stress, causing genomic instability. Oncogene-induced DNA damage, as one of the cancer-intrinsic features of damage, offers a possible basis for conditional synthetic lethality. Nuclear enzyme poly (ADP-ribose) polymerase 1 (PARP1) is best known for detecting single-stranded DNA breaks and affecting the process of DNA repair and transcription regulation. PARP1 is activated by zinc finger motif-mediated nicked DNA recognition. The ADP-ribosyltransferase catalytic domain of PARP then actuates the PARylation of PARP1 substrate proteins, mediating the recruitment of single-strand break repair (SSBR) machinery for DNA repair. Finally, PARP1 autoPARylation causes the release of PARP1 from DNA to another location for the next cycle of the SSBR process (54, 55). PARP inhibitors (PARPis), primarily designed for the catalytic site to interfere with DNA repair, cooperate with the pre-existing defects in homologous recombination (HR) repair to induce tumor cell death (56, 57). The oral PARPi olaparib is approved for the treatment of patients with metastatic breast cancer and a germline (g) *BRCA* mutation (58, 59). Recently, the last clinical trial of HER2-negative early breast cancer with (g) *BRCA* mutation indicated the significant efficacy of prolonging invasive and distant disease-free survival (60). Furthermore, the TBCRC 048 trial has expanded the clinical indications of PARPis for metastatic breast cancer patients with somatic (s) BRCA mutations or g/s mutations in HR-related genes such as g*PALB2* other than *BRCA*1/2 (61). In 2015, it was reported that ARID1A deficiency makes cancer cells more sensitive to PARPis, providing an innovative treatment strategy for patients with ARID1A-mutated breast tumors (62). Conversely, the team of Sourav found that ARID1A loss makes breast cancer insensitive to PARP inhibitors (63). This is probably related to the use of different breast cancer cell lines; therefore, primary cancer cells from patients will be used to further resolve this paradoxical phenomenon.

Ataxia-telangiectasia-mutated and Rad3-related protein kinase (ATR), a member of the phosphatidylinositol 3-kinase-like kinase family, is a central regulator of the cellular DNA damage response (64–67). In response to DNA damage, ATR can recruit ARID1A to DNA double-strand breaks for processing to single-strand ends and maintaining DNA damage signaling. A preclinical study indicated that the combination of ATR inhibitors and Wee1 inhibitors can increase DNA damage and suppress breast cancer metastasis (68). Meanwhile, ARID1A was identified as an ATR synthetic lethal partner. The inhibition of ATR disrupts the cell cycle, genomic stability, and cell survival in ARID1A-deficient tumor cells. Consequently, ATR inhibitors are also deemed to be a potential single-agent therapy for ARID1A-mutated cancers, including breast cancer (69).

### Epigenetic Regulation—BET and HDAC

Bromodomain and extraterminal domain (BET) proteins are chromatin readers that recognize acetylated proteins by a conserved bromodomain to regulate gene transcription (70, 71). BET proteins essentially contain two tandem bromodomains (BD1 and BD2), an extraterminal domain (ET), and a C-terminal domain (CTD). The ET domain recruits effector proteins for its regulatory function (72). The CTD interacts with positive transcription elongation factor b and promotes the phosphorylation of serine residues of the RNA Pol II C-terminal motif for transcriptional activation (73, 74). BET proteins consist of the ubiquitously expressed BRD2, BRD3, and BRD4 and the testis-restricted BRDT (75).

Previous studies have revealed that BRD4 plays key roles in embryogenesis and metastasis in breast cancer. BRD4 can regulate the self-renewal of mouse embryonic stem cells by promoting *Nanog* expression (76), and its loss causes early post-implantation defects in mice (77). In breast cancer, the long isoform BRD4 is a negative predictor of cancer metastasis and survival (78, 79), but unexpectedly, the short isoform BRD4 can induce EMT transition, CSC-like properties (79), migration, and metastasis through Engrailed-1-mediated enhancer regulation (80). BRD4 is considered a therapeutic target of tamoxifen-resistant breast cancer (81) because it participates in the regulation of ERα function (82, 83). In basal-like breast cancer, BRD4 links nonhistone Twist diacetylation to tumorigenesis (84). Moreover, there is a bromodomain-independent effect of BRD4 in BET-resistant TNBC cells (85). Given the importance of BRD4 in breast cancer, BRD4 is regarded as a therapeutic target by interacting with the bromodomain (86, 87) or proteolysis targeting chimeric (PROTAC) molecule (88–90). More importantly, ARID1A deficiency makes ER^+^ breast cancer cells sensitive to BET inhibitors (28). Although BET inhibitor resistance has become a challenging issue, synergy with multiple epigenetic agents, particularly histone deacetylase inhibitors, is expected to overcome this problem (91, 92).

HDAC1 protein is associated with the breast cancer stem cell phenotype and distant metastasis (93, 94). Reduced levels of BRMS1L (breast cancer metastasis suppressor 1 like) activates Wnt signaling receptor FZD10 expression by regulating HDAC1 recruitment and histone H3K9ac levels to promote breast cancer cell invasion and migration (94). In addition, it has been reported that HDAC1 participates in *ARID1A*-mediated gene expression. *ARID1A* loss inhibits HDAC1 recruitment and promotes H4ac-mediated BRD4 binding for gene transcription and growth (28). Because *ARID1A* mutations are mainly present in metastatic tumors or tumors progressing under endocrine therapy, a synthetic lethality-based endocrine treatment strategy with BET and/or HDAC1 inhibitors is expected to be used for *ARID1A*-mutated breast cancer (32).

### Residual SWI/SNF Activity—ARID1B

Mammalian SWI/SNF can be mainly composed of canonical Brahma-related gene 1 (Brg1)-associated factor (BAF) complexes and polybromo Brg1-associated factor (PBAF) complexes (95, 96). Brg1 and homologous Brahma (Brm) are the central ATPase subunits in SWI/SNF complexes (97). In addition to the shared ATPase Brg1/Brm, the SWI/SNF family also contains variant subunits and complex-specific subunits. ARID1A (BAF250A) and ARID1B (BAF250B) subunits are distinct from BAF complexes in a mutually exclusive fashion (15) replaced by ARID2 (BAF200) in PBAF complexes (98).

Considering that ARID1A mutations such as nonsense and frameshift usually result in loss-of-function traits, this defective protein is not suitable for targeting. ARID1A loss may make cancer cells more dependent on ARID1B-associated BAFs. Depletion of ARID1B destabilizes the SWI/SNF complex and suppresses the proliferation of ARID1A-deficient breast cancer (99). Meanwhile, the synthetic lethality between ARID1A and ARID1B has also been found to be a conserved function in colorectal cancer (100), ovarian clear cell carcinomas (101), and ovarian cancer (102) with an ARID1A-mutant background. Hence, ARID1B may be a promising target for the potential treatment of ARID1A-mutated cancers.

Previous studies have revealed that ARID1A, ARID1B, and ARID2 contain some mutations and are inactivated in recurrent breast cancer (29). In TNBC, ARID1B expression was upregulated and related to worse clinical prognoses (103, 104). In view of the importance of ARID1B for facilitating BAF ATPase module binding to chromatin, approaches with small stabilized peptides can be considered for disrupting the association of ARID1B with the BAF core module (105), particularly SMARCC and SMARCD (99). A homologous approach has been used to interfere with the enhancer of zeste homologue 2 (EZH2)/EED complex in EZH2-dependent cancer (106). BET-PROTACs were used to target and degrade BRD4 protein to revert BETi resistance (90). These strategies can be employed for ARID1B treatment. Hence, strategies of synthetic lethality between ARID1A and ARID1B or ARID2 are worthy of further exploration.

### Tumor Immunological Microenvironment

Previous preclinical and clinical studies have reported that ARID1A mutation can enhance the immunogenicity of tumor cells in breast cancer (107) and improve tumor sensitivity to immune checkpoint inhibitors in various cancers, including metastatic urothelial cancer (108), advanced nonsmall cell lung cancer (109), ovarian clear cell cancer (110), colorectal cancer (111, 112), gastrointestinal cancer (113), and colon cancer (114), which can provide insights into ARID1A-mutated breast cancer. Meanwhile, ATM inhibitors and HDAC6 inhibitors can further potentiate the efficacy of antitumor immunity in ARID1A-mutated ovarian cancer (115, 116). The efficacy of immune checkpoint inhibitors in ARID1A-mutated metastatic breast cancer is expected to be good.

### Glutathione Metabolic Pathway

A recent study showed that ARID1A-deficient cancer cells are specifically vulnerable to inhibitors of the glutathione (GSH) metabolic pathway due to lower GSH levels (117). Furthermore, downregulation of GSH was associated with poor prognosis in breast cancer (118). GSH is also considered a potential therapeutic target for metastatic breast cancer, including metastatic brain colonization and breast cancer bone metastasis (119–121). Consequently, targeting the GSH metabolic pathway may offer hope for breakthroughs in the fight against ARID1A-deficient metastatic breast cancer.

### EZH2 Inhibition

Furthermore, EZH2 is also regarded to be specifically vulnerable in ARID1A-mutated cancers (122). EZH2 is a core member of the polycomb repressive complex 2 (PRC2) and is responsible for catalyzing H3K27me3, which is associated with transcriptional repression (123, 124). EZH2 cooperates with ARID1A to regulate *PIK3IP1* expression and the PI3K/AKT signaling pathway, and EZH2 inhibitors can reduce the proliferation of ARID1A-mutated tumors. Given that ARID1A-deficient tumors are sensitive to both tumor immunotherapy and PARP inhibitors, the combination of EZH2 inhibitors with PARP inhibitors or tumor immunotherapy with ARID1A-deficient tumors may be potential treatment strategies (62, 125, 126). EZH2 expression levels or protein modifications are involved in breast cancer initiation (127–129), progression or metastasis (130–133), breast cancer stem cell regulation (134), and drug resistance (135–141). The high expression level of EZH2 is also considered an indicator of aggressive breast cancer (128). The MEK–ERK pathway (142) and lncRNA ANCR (143) can regulate EZH2 levels to impact breast cancer metastasis. Meanwhile, EZH2 undergoes post-translational modifications, including arginine methylation, lysine methylation (144), and serine/threonine phosphorylation (145), regulating protein methyltransferase activity (146), protein stability (147), or cellular localization (148), which ultimately affect cancer metastasis. Considering the crucial role of EZH2 in the process of breast cancer metastasis and endocrine resistance (138), EZH2 inhibition represents a novel treatment option for ARID1A-mutated metastatic breast cancer.

## Conclusion and Discussion

Cancer metastasis, as the paramount factor in cancer-related death, places both an economic and psychological burden on breast cancer patients. Previous studies on genomic alterations acquired during metastatic evolution highlight the value of molecular features for therapeutic strategies and have contributed to the substantial improvement in cancer outcomes. Recently, *ARID1A*, a subunit of the SWI/SNF complex, was found to be commonly mutated in recurrent breast cancer and is considered a promising but challenging drug target. Inactivated ARID1A mutations are important to maintain the luminal identity of breast cancer and are associated with endocrine resistance. Based on synthetic lethality and cellular dependence, therapeutic strategies targeting ARID1A-mutated breast cancer have been reported, such as some inhibitors of PARP, EZH2, and BRD4. Moving forward, further mechanistic studies and clinical trials are needed to determine the efficacy of cancer targets driven by *ARID1A* mutations.

There is still much confusion regarding ARID1A mutations and metastatic breast cancers. Although we know that ARID1A is the most commonly mutated gene in SWI/SNF complexes and has a higher incidence in metastatic breast cancer, the functions and mutation frequencies of ARID1A in different subtypes of breast cancer are not clear. Whether the occurrence of ARID1A-mutated metastatic breast cancer has an organ tendency needs more statistical analysis. There are more potential therapeutic targets that have been discovered and proven effective in preclinical studies, but the clinical trial outcomes are limited, and the underlying mechanisms are not fully understood. Indeed the responses of BET inhibitors in TNBC are exactly the opposite of those in ER^+^ breast cancer (149). In addition, the indications and the combination strategies of different therapeutic targets remain key questions and active areas of research. CDK4/6 inhibitors (150) or BRD4 inhibitors (151, 152), for example, drive antitumor immunity and provide a reasonable combination strategy with immunotherapies, while CDK4/6 may interfere with the activity of ATR-associated drugs that rely on replicative stress (153). Consequently, more investigations of ARID1A-mutated breast cancer are necessary and expected.

## Author Contributions

XuaC, J-XZ, X-CC, and FD prepared and reviewed the manuscript. All authors contributed to the article and approved the submitted version.

## Conflict of Interest

The authors declare that the research was conducted in the absence of any commercial or financial relationships that could be construed as a potential conflict of interest.

## Publisher’s Note

All claims expressed in this article are solely those of the authors and do not necessarily represent those of their affiliated organizations, or those of the publisher, the editors and the reviewers. Any product that may be evaluated in this article, or claim that may be made by its manufacturer, is not guaranteed or endorsed by the publisher.
